# Central Obesity and Coronary Heart Disease Risk Factors in Referral Outpatients to Zahedan Cardiology Clinic, Iran

**DOI:** 10.5812/ijhrba.4275

**Published:** 2012-07-25

**Authors:** Mansour Shahraki, Touran Shahraki, Bahram Pourghasem Gargari, Nourallah Ramroudi

**Affiliations:** 1 Children and Adolescent Health Research Center, Zahedan University of Medical Sciences, Zahedan, IR Iran; 2 Department of Nutrition, Zahedan University of Medical Sciences, Zahedan, IR Iran; 3 Department of Pediatrics, Faculty of Medicine, Zahedan University of Medical Sciences, Zahedan, IR Iran; 4 Department of Nutrition and Biochemistry, Faculty of Health and Nutrition, Tabriz University of Medical Sciences, Tabriz, IR Iran; 5 Nutrition Research Center, Tabriz University of Medical Sciences, Tabriz, IR Iran; 6 Department of Neurology, Faculty of Medicine, Zahedan University of Medical Sciences, Zahedan, IR Iran

**Keywords:** Central Obesity, Coronary Artery Disease, Risk Factors, Iran

## Abstract

**Background:**

Coronary Heart Disease (CHD) is one of the most common and life-threatening diseases in both developed and developing countries and a close correlation has been found between different types of obesity and CHD.

**Objectives:**

The current study investigated the relationship between central obesity and coronary heart disease risk factors in CHD referral outpatients to Khatam Clinic, Zahedan, Iran.

**Patients and Methods:**

In this clinical, cross-sectional study, data for 120 CHD patients aged 30–60 years were included. Based on waist-to-hip ratio (WHR), subjects were classified into two groups: not centrally obese (NCO; WHR ≤ 0.95 for men, ≤ 0.8 for women) and centrally obese (CO; WHR > 0.95 and > 0.8 for men and women, respectively). Triglyceride (TG), total cholesterol (TC), blood urea nitrogen, creatinine, and fasting blood sugar (FBS) were enzymatically determined. Sitting systolic and diastolic blood pressures were measured for each patient.

**Results:**

Significantly more women than men and significantly more subjects with less education than subjects with more education were CO. Rates of CO were higher in subjects 45–60 years old than in those 30–45 years old (n.s.). CO subjects also had higher mean systolic and diastolic blood pressure, TG, and creatinine levels (n.s.). Significantly higher mean values were observed for FBS in CO subjects than in NCO subjects (P = 0.02). Mean values for smoking were significantly higher in the NCO group compared with the CO group (P = 0.004).

**Conclusions:**

According to the results of this study, in CHD patients, central obesity was associated with certain risk factors, especially FBS. Central obesity was more evident in women, less educated subjects, and older subjects. Further research is required to clarify these associations.

## 1. Background

One of the most common and life-threatening diseases in both developed and developing countries is coronary heart disease (CHD) (1). A close correlation has been found between different types of obesity and CHD (2). Various anthropometric measurements have been used for interpretation of obesity, one of which is central obesity. Central obesity is closely associated with intra-abdominal fat and is measured either by waist circumference or waist-to-hip ratio (WHR) (3). Overweight and obesity are associated with some lipoprotein disturbances (4). The results of some studies in both sexes and different age groups have shown that percentage of body fat is positively correlated with total cholesterol (TC), triglycerides (TG), and low-density lipoprotein cholesterol, and negatively associated with high-density lipoprotein cholesterol (5). The results of other studies have shown that the risk of central obesity increases with age (6), especially in women > 50 years old (7). Some studies have indicated an inverse relation between level of education and obesity (8), but other studies found that body fat distribution, as assessed by WHR, was not related to education level in either sex (9). An association between central obesity and higher TG has been demonstrated (10). Some researchers have also identified an association between hypertension, FBS, and centrally located body fat (11, 12). The effect of smoking on WHR is under debate. Some studies (13) reported a weak but significant association between smoking and WHR, while others failed to show such an association (14). Although information is lacking about the relationship between WHR and parameters of renal function, Koc et al. (15) demonstrated a significant relationship between creatinine levels and WHR. Due to the scarcity of data on and controversy about the relationship between WHR and various parameters, this study evaluated the relationship between risk factors of CHD in subjects with different WHR.

## 2. Objectives

The current study investigated the relationship between central obesity and coronary heart disease (CHD) risk factors in CHD referral outpatients at Khatam Clinic, Zahedan IRAN.

## 3. Patients and Methods

### 3.1. Study Subjects and Geographical Area

Data were collected from one public cardiology clinic in Khatam Hospital in Zahedan, Sistan and Balouchestan Province, Iran. In this clinical, cross-sectional study, data for 120 CHD outpatients (62 men and 58 women) from 30–60 years of age who had referred to the clinic were studied. On the basis of WHR, the subjects were classified into two groups: not centrally obese (NCO; WHR ≤ 0.95 for men, ≤ 0.8 for women) and centrally obese (CO; WHR > 0.95 and > 0.8 for men and women, respectively). Patients completed a questionnaire inquiring about age, job, and level of education. This study was approved by the research committee of the medical faculty of Zahedan University of Medical Sciences. Informed consent obtained from all patients.

### 3.2. Anthropometric Measurements

All anthropometric measurements were made by trained staff in accordance with WHO standards (16). Waist circumference (waist and hip girth) was measured to the nearest 0.5 cm using a fiberglass butterfly tape placed midway between the costal margin and the iliac crest in the horizontal plane. Hip circumference was measured at the point over the buttocks yielding the maximum circumference. WHR was calculated as waist circumference (cm) divided by hip circumference (cm).

### 3.3. Inclusion and Exclusion Criteria

CHD cases in the present study were required to have been diagnosed with and experienced the following: myocardial infarction, angina pectoris, coronary artery bypass graft, ischemic changes on electrocardiography, and positive angiography. Subjects < 30 or > 60 years of age and pregnant women, and those with chronic renal failure, infection, inflammation, diabetes mellitus type 2, hyperlipidemia, thyroid function disorders, secondary obesity (due to hypothyroidism or Cushing syndrome), and some chronic illnesses were excluded.

### 3.4. Evaluation of Coronary Heart Disease Risk Factors

Coronary heart disease risk factors examined in the current study included FBS, TC, TG, systolic and diastolic blood pressure, and smoking. Blood urea nitrogen and creatinine were also measured. For evaluation of these risk factors, blood samples were drawn with subjects in a sitting position after a 12-hour overnight fast between 7:00 and 8:00 a.m. Samples were centrifuged within 30–45 min of collection. All assays were performed at the hospital laboratory 1 hour after sample collection. TC and TG were assayed using enzymatic colorimetric tests (TC and TG Kits, Pars Azmoon, Inc., Tehran, Iran). FBS level was measured using enzymatic methods (Pars Azmoon).

### 3.5. Blood Pressure, Smoking and Education

Blood pressure in sitting position was measured using a mercury sphygmomanometer model (Riester, Germany) after a 5-min rest. Systolic (Korotkoff phase 1) and diastolic (Korotkoff phase 5) blood pressure were measured twice on the upper left arm, and the average of the two measurements was used for analysis. In addition, subjects were asked to state the number of cigarettes smoked per day and categorized into two groups, accordingly: smokers and nonsmokers. Data analysis also included two levels of education: subjects with 1–12 years of schooling and those with > 12 years of schooling.

### 3.6. Statistical Analysis

All statistical analysis was performed using the SPSS/PC statistical program (version 13 for Windows; SPSS, Chicago, IL, USA). Student’s t-test was used for determination of differences between variables. The χ^2^ and Fisher’s exact test were used to determine correlations between age, sex, education level, and WHR. All values were presented as Means ± Standard deviations. A value of P < 0.05 was considered statistically significant.

## 4. Results

The results showed that 69 (57.5%) CHD patients were CO and 51 (42.5%) were NCO (n.s.). Correlation testing revealed that significantly more women than men and significantly more subjects with less education than subjects with more education were CO. Rates of CO were higher in subjects 45–60 years old than in those 30–45 years old (*Table 1*). Student’s t-test revealed higher mean values in CO subjects for systolic and diastolic blood pressure, TG, and creatinine (n.s). Significantly higher mean values were observed for FBS in CO subjects than in NCO subjects (P = 0.02), (*Table 2*). The number of cigarettes per day was significantly higher in the NCO group compared with the CO group (P = 0.004). No significant difference was observed between the two groups in TC (*Table 2*).

**Table 1.** Comparison of Demographic Data

	NCO ^a^, No. (%) (n = 51)	CO ^a^, No. (%) (n = 69)	*P* value
**Age, y**			0.34
30–45	11 (52)	10 (48)	
45–60	40 (40)	59 (60)	
**Sex**			0.0005
Male	49 (79)	13 (21)	
Female	2 (3.4)	56 (96.6)	
**Literacy level**			0.0005
1-12	32 (68)	15 (32)	
≥ 12	19 (26)	54 (74)	

^a^ Abbreviations: NCO, centrally non-obese; CO, centrally obese

**Table 2.** Some Blood Parameters, Systolic and Diastolic Blood Pressures, Blood Urea Nitrogen, Creatinine and Smoking in Centrally Obese and Non-Centrally Obese Subjects

	NCO ^a^, Mean ± SD (n= 51)	CO ^a^, Mean ± SD (n= 69)	*P* value
Fasting blood sugar, mg/dL	118 ± 35	143 ± 74	0.024
Total cholesterol, mg/dL	202 ± 55	199 ± 71	0.83
Triglyceride, mg/dL	143 ± 68	150 ± 79	0.63
Blood urea nitrogen, mg/dL	17.75 ± 8.41	17.96 ± 6.45	0.87
Creatinine, mg/dL	0.95 ± 0.29	1.01 ± 0.3	0.32
Systolic blood pressure, mmHg	117 ± 22	122 ± 22	0.17
Diastolic blood pressure, mmHg	73 ± 15	76 ± 15	0.31
Smoking (cigarettes per day)	8 ± 16	2 ± 4	0.004

^a^ Abbreviations: NCO, centrally non-obese; CO, centrally obese

## 5. Discussion

### 5.1. Waist-to-Hip Ratio, Gender and Education

The results of this study revealed that a significantly higher proportion of CO subjects were women. Similar findings were observed in studies conducted in other parts of Iran (17). The high percentage of CO female subjects may be related to lower activity levels. Women in Sistan and Balouchestan Province lack exercise due to religious and cultural customs. Research has shown that Iranian people, especially women, have inadequate levels of physical activity (18). In addition, in Iranian culture, obesity is considered a sign of health and beauty. This attitude may contribute to current high rates of CO in Iran. The results of the current study showed that CO was significantly more prevalent in subjects with less education than those with more education. This finding is similar to those of some other studies (19), although results are inconsistent (9, 20). In the region of Iran examined here, higher education leads to better employment, which may correlate with increased physical activity and lower rates of central obesity. Further study is required to verify this possibility. Research has also shown that education level may affect quality of diet (21).

### 5.2. Waist-to-Hip Ratio and CHD Risk Factors

Student’s t-test in this study revealed that CO subjects had slightly higher TG levels (n.s.) and significantly higher (P = 0.02) mean values of FBS compared with NCO subjects. Central obesity indices have been shown to be associated with high TG concentrations (10). An association between body fat distribution and FBS has also been reported in some studies. Neri et al. (12) showed an increased risk of FBS levels with centrally located body fat. In this study, higher mean values for FBS in the CO group than in the NCO group may be due to high carbohydrate consumption, especially bread and other cereals. These foods constitute the main source of energy in various parts of Iran (22). Central obesity increases risk of hypertension (11). The data in this study revealed an insignificant increase in mean systolic and diastolic blood pressure values in CO subjects compared to NCO subjects. Similar to the results of this study, Rodrigues Barbosa et al. (23) found no association of systolic blood pressure and WHR. Doll et al. (24) showed an increased risk for hypertension with increased WHR. The mean number of cigarettes smoked per day was significantly higher (P = 0.004) in the NCO group than in the CO group. Accumulation of body fat in the abdominal region may be the consequence of behavioral factors such as smoking (25). Smoking is considered one of the confounding factors in evaluating the relationship between fat distribution and CHD risk profile. In the present study, an inverse significant association between smoking and WHR was observed, similar to that in other studies (26). The relationship between smoking and appetite is well known. In conclusion, according to the results of this study, among CHD patients, no significant differences were observed between CO and NCO patients. Among the CHD risk factors, only FBS was significantly higher in CO subjects than in NCO CHD patients. Central obesity was more prevalent in women and less educated subjects. In this study, high-density lipoprotein cholesterol was not measured, so Framingham’s point score could not be calculated. Other factors which could affect blood lipoproteins, such as sex hormones and dietary habits, were not measured in our study. Future research will fill these gaps and contribute further to our understanding of the relationship between central obesity and CHD risk factors.
